# Paternal Age Alters Social Development in Offspring

**DOI:** 10.1016/j.jaac.2017.02.006

**Published:** 2017-05

**Authors:** Magdalena Janecka, Claire M.A. Haworth, Angelica Ronald, Eva Krapohl, Francesca Happé, Jonathan Mill, Leonard C. Schalkwyk, Cathy Fernandes, Abraham Reichenberg, Frühling Rijsdijk

**Affiliations:** a Social, Genetic and Developmental Psychiatry (SGDP) Centre, King's College London, UK; b Seaver Autism Center for Research and Treatment, Icahn School of Medicine at Mount Sinai, New York; c University of Exeter Medical School, University of Exeter, Exeter, UK; d MRC Integrative Epidemiology Unit, School of Experimental Psychology and School of Social and Community Medicine, University of Bristol, UK; e Birkbeck University of London, UK; f School of Biological Sciences, University of Essex, Colchester, UK

**Keywords:** advanced paternal age, social development, autism, schizophrenia, neurodevelopment

## Abstract

**Objective:**

Advanced paternal age (APA) at conception has been linked with autism and schizophrenia in offspring, neurodevelopmental disorders that affect social functioning. The current study explored the effects of paternal age on social development in the general population.

**Method:**

We used multilevel growth modeling to investigate APA effects on socioemotional development from early childhood until adolescence, as measured by the Strengths and Difficulties Questionnaire (SDQ) in the Twins Early Development Study (TEDS) sample. We also investigated genetic and environmental underpinnings of the paternal age effects on development, using the **A**dditive genetics, **C**ommon environment, unique **E**nvironment (ACE) and gene–environment (GxE) models.

**Results:**

In the general population, both very young and advanced paternal ages were associated with altered trajectory of social development (intercept: *p* = .01; slope: *p* = .03). No other behavioral domain was affected by either young or advanced age at fatherhood, suggesting specificity of paternal age effects. Increased importance of genetic factors in social development was recorded in the offspring of older but not very young fathers, suggesting distinct underpinnings of the paternal age effects at these two extremes.

**Conclusion:**

Our findings highlight that the APA-related deficits that lead to autism and schizophrenia are likely continuously distributed in the population.

Advanced paternal age (APA) at conception has been linked to a number of negative neuropsychiatric outcomes in offspring, including low academic achievement,^1^ hyperactivity,^1^ and suicide.^2^ A relation with autism and schizophrenia has been most robustly replicated in epidemiological studies.^3^, ^4^, ^5^, ^6^ Odds ratios for these disorders were shown to increase in men more than 35 years old at conception, with even higher risk observed in older groups of fathers. Men in their 40s at conception have been shown to have two to three times higher chances of conceiving a child with either autism or schizophrenia,^3^, ^6^, ^7^, ^8^, ^9^, ^10^ raisings concerns about recent increases in age at parenthood. Although several studies further indicated that such effects likely persist through generations,^11^, ^12^ the evidence remains conflicting.^13^

Currently, there is little consensus regarding whether the effects of APA arise due to de novo genetic mutations that accumulate in paternal spermatogonia with age,^14^, ^15^ or familial factors.^16^, ^17^, ^18^ Proponents of the latter hypothesis postulate that men who conceive at an advanced age are likely to do so because of their social difficulties, and therefore themselves display mild autism/schizophrenia traits. Under this scenario, higher rates of autism/schizophrenia among offspring of older fathers are mainly due to stable (age-independent) characteristics of men who delay fatherhood.

Given that both autism and schizophrenia affect normal development^19^, ^20^ and are characterized by severe social impairments, as well as arise due to partly overlapping genetic influences,^21^, ^22^ robust association between these disorders and APA may help guide research into mechanisms vulnerable to the APA effects. It has been hypothesized^23^ that the increased risk for autism and schizophrenia in offspring of older fathers is mediated, at least in part, by the influence that APA exerts on social development.

Previous animal^24^ and human^23^ studies have documented an association between APA and social functioning; however, negative results have also been reported.^25^ Our group has recently extended this line of research by using murine models to show that the effects of APA on offspring social behavior are developmentally dynamic,^26^ meaning, compared to mice born to fathers at a standard breeding age, those with older fathers were characterized by hypersociability early in life, and hyposociability upon reaching adulthood. Nevertheless, in contrast to a high number of publications linking APA with outcomes measured at a single time point (e.g., Sandin *et al.* and Brown *et al.*^8^, ^9^), studies of developmental trajectories in relation to paternal age at conception have been lacking to date. This is despite their methodological adequacy: if the offspring of older fathers show a developmental delay but can compensate for it later in life, such patterns could be missed in studies that considered their outcomes at only one time point. Moreover, differences in the social functioning observed between people with autism and the general population are likely dynamic, resulting from disruptions in the process of acquisition/refinement of some social skills, for example, theory of mind,^27^ and such developmental nature should be reflected in the design of studies exploring origins of this disorder.

Here we hypothesized that the same mechanisms that link APA with the risk of developmental disorders could also interfere with normal maturation processes across the behavioral traits underlying these conditions. In other words, offspring of older fathers could display developmental differences akin to, but less severe than, those observed in clinical cases (Figure 1). This would be in line with the model postulating that psychiatric disorders represent quantitative extremes of normally distributed traits.^28^, ^29^, ^30^, ^31^ For example, various degrees of autistic-like social impairment are present across the entire population, and the trait is thought to be only quantitatively, rather than qualitatively, different when displayed by cases and controls.^32^ Both quantitative^33^ and molecular genetics studies^34^ suggest overlapping genetic effects on diagnosed autism and individual differences in subclinical autism traits.

The present study uses a population-based, nationally representative cohort of twins (Twins Early Development Study [TEDS]^35^) to examine the effects of paternal age on the developmental trajectories in children. Lundström *et al.*^36^ previously showed that, in the same sample, the APA effects were observed in a nonclinical population at the offspring age of 9. Our design allows us to trace the development of these effects, examining how early such effects emerge, as well as their subsequent life course. Although paternal influences were of primary interest in this study, the TEDS sample includes detailed background information that also enabled us to assess how behavioral changes over childhood are affected by maternal age at conception, controlling for other important confounders. This allowed us to tease out independent contributions of parental ages to offspring development, while controlling for other demographic factors.

In line with our hypothesis, the focus remained on the effects of APA on social development. However, to explore possible specificity of these influences, we also analysed other behavioral dimensions, including hyperactivity, conduct, peer problems, and emotionality. Although these domains strongly affect social functioning, and have been shown to be altered in subpopulations of individuals with autism and schizophrenia,^37^, ^38^, ^39^ they do not class as core symptoms of either of these disorders. Finally, the twin structure of our sample allowed us to indirectly test the competing hypotheses regarding de novo versus familial genetic influences driving the effects of APA.

## Method

### Sample

The study used the TEDS sample, a large nationally representative cohort of British twins.^35^ The total sample consists of more than 15,000 twin pairs with detailed phenotypic data, collected longitudinally. Measures relevant for social functioning were assessed in these individuals around the ages of 4, 7, 9, 14, and 16 years. Exclusion criteria for our analyses were as follows: (1) confirmed autism diagnosis, assessed at age 12 years through the Development and Well-Being Assessment^40^ (DAWBA), which was shown to have high sensitivity (0.88) and specificity (0.85) for ASD^41^ in the TEDS sample; (2) other medical exclusions/being a perinatal outlier (any of: birth weight <471 g, >97 days of special care after birth, >74 days of hospital stay after birth, born before 27th week of gestation, maternal alcohol drinking of >14 units per week during pregnancy); and (3) uncertain zygosity—all standard exclusions for this cohort and for community samples. We decided to exclude the individuals with autism to limit the possibility that the association between paternal age and social development in our study is driven by higher rates of the diagnosis in the older paternal age categories (previously shown in the TEDS sample^36^). As we recognized that by doing so we might have truncated part of the social development continuum, all analyses were also run in the full sample.

### Measures

Social development was investigated using parents’ (predominantly mothers’) ratings of the Strengths and Difficulties Questionnaire (SDQ).^42^ The SDQ was designed to provide a succinct tool for assessing both good and problematic aspects of behavior in children aged 4 to 16 years. The five subdomains include conduct problems, emotional symptoms, hyperactivity, peer problems, and prosocial behavior. In line with the goals of the current study, the prosocial subdomain was of main interest in our analyses; however, we tested the effects of paternal age on all SDQ dimensions to investigate the specificity of possible effects on social development. Each subdomain is assessed through five questions scored from 0 to 2, so that the maximum score in each subdomain is 10; unlike the other subdomains, high scores in prosocial domain indicate less problematic behavior. The data in the current study came from years 4, 7, 9, 12, and 16 data collection waves, with only prosocial items available for the last time point.

### Statistical Analysis

All analyses were performed using R version 3.1.0^43^, OpenMX,^44^ and STATA 14.

To create paternal age categories (PAC) used for obtaining paternal age−specific behavioral trajectories, we used the age range cut-offs specified by Lundström *et al.* (2010)^36^: <25, 25 to 34, 35 to 44, 45 to 50, and >51 years (PAC 1−5 levels). Age, sample size, maternal age, socioeconomic status (SES) and sex composition were recorded for each paternal age group. Given that there were few much older mothers in our sample, the categorization for the maternal age differed: <20, 21 to 30, 31 to 40, and >40 years (maternal age category [MAC] 1–4 levels). Correlations between maternal and paternal ages were estimated using Pearson’s product−moment correlation coefficient.

### Multilevel (Mixed) Models for Longitudinal Data

The developmental change in the SDQ scores can be described in terms of their intercept and slope (growth curve coefficients). Whereas the intercept reflects the mean group value at the starting point, the slope reflects the developmental growth. For example, higher initial sociability was indicated by high values of the prosocial intercept, and increase in sociability over time by its positive slope. Significant effects of paternal age on the slope coefficients would suggest that social development is influenced by father’s age at conception. Using longitudinal data from a twin registry, we adjusted for the within-family and within-individual correlation of the data by using multilevel (/mixed) modeling (R package *lme4*). Details of the model specification are provided in the supplementary material, available online.

In all adjusted analyses, we controlled for the effects of age of the other parent, SES (index of parent qualifications and employment, and mother’s age at birth of first child), sex, and zygosity. We investigated the effects of parental age as both a categorical and continuous variable. Given that the former necessitated multiple pairwise comparisons, we controlled for multiple testing using the false discovery rate (FDR) method (all *p* values present postadjustment scores). To obtain smooth curves based on the results of these models, we used locally weighted scatterplot smoothing.

### Twin Model−Fitting Analysis

In the second stage, we investigated the extent to which individual differences in growth curve coefficients (intercept and slope) could be explained by latent genetic and environmental effects, as well as how these effects change with paternal age.

Our prediction was that the degree to which the between-individual variance in social development could be attributed to environmental and genetic factors would change across paternal age range. We hypothesized that developmental patterns would be affected primarily by environmental factors in the offspring of younger fathers, with these factors having little impact on children born to middle-aged and older men. Conversely, our prediction was that genetic sources of variance would become more prominent in the offspring of older fathers. To investigate these assumptions, we tested nonlinear (quadratic) effects of paternal age on genetic and environmental variance components. To retain focus on the paternal age effects and to avoid multiple testing, twin modeling on the slope and intercept scores was performed in relation to paternal but not maternal age, and only on the SDQ domains where we observed significant effects of paternal age in multilevel models.

As for the growth curve modeling, paternal age was treated as either a categorical (Additive genetics, Common environment and unique Environment [ACE] analysis) or continuous (gene–environment [GxE] analysis) moderator in the twin analyses. Both methods are described below, and further detail regarding these models can be found in the supplementary material, method, and Tables S1 to S20, available online.

#### ACE Analysis

ACE analysis allows us to disentangle contribution of genetic and environmental factors to the trait variance. All analyses were run in both the full sample and stratified by paternal age (PAC 1−4 categories; PAC4 and PAC5 groups were collapsed for these analyses because of insufficient number of individuals with very old fathers to perform twin analyses). While the full-sample analysis informs about the degree to which development parameters are heritable in the population, stratification by father's age at conception allowed us to observe whether the heritability estimates are stable across different paternal age groups. For all analyses, we used the slope and intercept scores, computed individually for all twins. To obtain standardized genetic and environmental variance components, we ran ACE and AE models for the SDQ coefficients. The standardized A, C, and E components are referred to as h^2^, c^2^, and e^2^. (For more details on these models, see Rijsdijk and Sham^45^ or Neale and Maes.^46^)

#### GxE Analysis

To gain further insights into the potential moderating effects of paternal age on the genetic and environmental variance components, we also ran a GxE model. This allowed us to investigate whether the degree to which genetic or environmental factors affect social development changes continuously with paternal age. To limit the possibility that the moderating effects of paternal age on these factors are due to genetic correlation between age at fatherhood and social development, paternal age effects were regressed out of the growth curve coefficients before fitting the GxE model. For all individuals with missing data on paternal age, the variable was imputed at the sample mean (33.37 years).

## Results

### Cohort Characteristics

There was a significant, positive correlation between paternal and maternal ages (*R* = 0.61, *p* < .001). Table 1 presents demographics of the sample stratified by paternal age group.

### Developmental Trajectories: SDQ—Prosocial Domain

#### Paternal Age as a Continuous Variable

In the developmental analysis, the quadratic model produced a better fit than the linear one, for both the intercept and the slope (intercept: χ^2^ = 9.60, df = 1, *p* < .001; slope: χ^2^ = 4.97, df = 1, *p* = .03). Effects of paternal age on both of the coefficients were significant in the crude (Table S1, available online) and adjusted models (intercept_adj_: β = 1.37E^−3^, *p* = .01; slope_adj_: β = −1.39E^−4^, *p* = .03). The shape of the association was an inverted U for the slope, and was U-shaped for the intercept coefficient (respectively, negative and positive quadratic estimates). This indicated that offspring of fathers at the extreme ends of paternal age distribution tended to have the highest initial sociability scores, but showed less developmental change than other groups. The same patterns were recorded in the full sample with no exclusions (Table S3, available online).

#### Pairwise Comparisons

**Figure 2:**
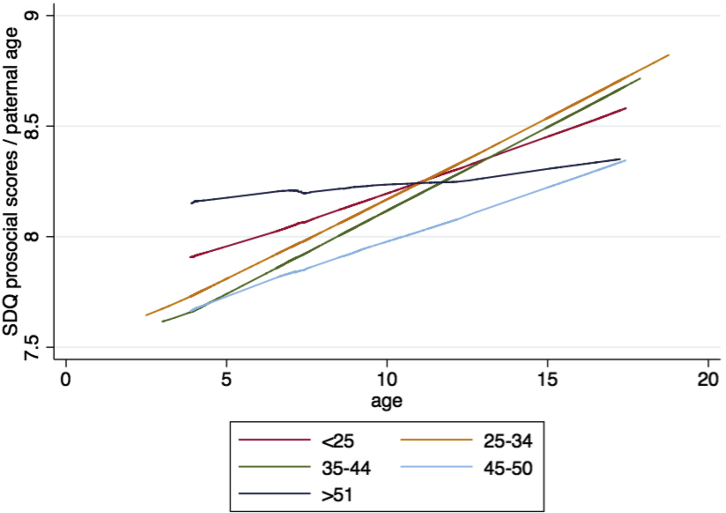
Trajectories of change in the Strengths and Difficulties Questionnaire (SDQ) prosocial scores over time in different parental age groups. Note: Graphs show results for the fully adjusted model, by paternal age categories.

Results were validated in the models using paternal age as a categorical variable (PAC1-5). Initial prosocial scores were highest in the offspring of the oldest fathers (PAC5), followed by the youngest paternal age group (PAC1). Over time, scores increased in all groups, but least so for the offspring of the youngest and oldest fathers, who, in the final assessment, were lagging behind offspring of fathers in their mid-20s to mid-40s at conception (Figure 2, Table 2). These results were exemplified by statistically significant differences in the intercepts and slopes presented in Table S4 (crude model) and Table S9 (fully adjusted model), available online. Analysis of the full sample (no exclusions) revealed the same patterns (Tables S17 and S18, available online).

Trend analyses and pairwise comparisons indicated no significant differences in either the intercept or the slope of the prosocial scores in relation to maternal age at conception.

### SDQ—Other Domains

Pairwise comparisons indicated that paternal age at conception was associated with differences in conduct problems, emotionality, and hyperactivity (Tables S4−S8, available online). These effects nonetheless disappeared in the adjusted model. Results from the full models indicated that young maternal age is associated with developmental differences in all of these domains. Offspring of younger mothers had higher hyperactivity and conduct, but lower emotionality scores in early development in comparison to mothers who were middle-aged at conception (Tables S12, S14, and S16, available online). Neither paternal nor maternal effects were significant in developmental analysis in which parental ages were treated as continuous variables.

### Paternal Age Effects on Heritability of Social Development

Given that the twin correlations did not suggest dominance effects (Table S19, available online), we fitted the ACE model for the prosocial scores coefficients. Shared environmental effects (C) were present in the analyses stratified by paternal age, but not in the full sample (Table S20, available online); therefore, we report ACE and AE models, respectively.

Analyses in the full sample suggested the variance in the prosocial scores intercept and the slope was influenced predominantly by genetic factors (Table 3). The stratified analyses revealed higher genetic and lower environmental influences on both the intercept and the slope variable in the offspring of the oldest fathers (PAC4); however, the difference between this and other groups was not significant (overlapping CIs).

GxE analyses indicated no significant moderating effects of paternal age on either genetic or environmental variance in social development.

## Discussion

The results of this study demonstrate that paternal age contributes to individual differences in social development in the general population, with no similar effects on other behavioral domains. To the best of our knowledge, this is the first time that a longitudinal approach was used to show such effects outside the clinical extremes. Our findings highlight that the effects of paternal age on social development may range from mild alterations in the typical maturation patterns to severe clinical phenotypes. This indicates that some children born to older fathers may experience difficulties in social settings, and the challenges that they face may increase as they get older, even if they do not meet any diagnostic criteria. Our suggestion that the mechanisms involved in social development are particularly vulnerable to the APA effects can help to explain the robust association between the APA and autism and schizophrenia, and creates new testable hypotheses regarding molecular underpinnings of the effects observed in our sample.

Our results also suggested that the genetic influences on social development parameters become more pronounced in the offspring of the oldest group of fathers, in line with our predictions that these effects likely follow a nonlinear trajectory. We could not, however, verify the de novo versus inherited nature of these effects in the GxE model, likely because of insufficient power to estimate such nonlinear effects in our sample.

### Trajectories of Social Behavior

Social development was the main focus of this study, as this domain is notably affected in the disorders with most established associations with APA.^23^, ^24^ To assess the social maturation curve, we analyzed the changes in the SDQ prosocial scores between the ages of approximately 4 and 16 years. To investigate whether paternal age effects are specific to prosocial domain or manifest themselves also in other behavioral dimensions, we also explored their influence on hyperactivity, emotionality, conduct, and peer problems.

Although all behavioral domains measured in the SDQ likely affect social functioning, psychometric data support a five-factorial structure of the SDQ, indicating the separateness of the behavioral constructs reflected in each of the subscales.^47^ It is therefore plausible to hypothesize that the SDQ domains will be underlain by different genetic and environmental factors, and therefore that APA could selectively affect only some of them. Although it was recommended that, in certain settings, using three- rather than five-factor structure (internalizing: emotionality and peer problems; externalizing: conduct and hyperactivity; and prosocial) may be preferential,^48^ prosocial behavior is considered a construct distinct from the other SDQ measures, even under this protocol.

Our analyses indicated that SDQ prosocial trajectories were significantly altered in relation to paternal age at conception. Individuals with the youngest and oldest fathers received the highest parental ratings for prosocial behavior early in life (indicated by high SDQ intercept scores). However, these groups showed the least developmental change, and by the end of the assessment period displayed less prosocial behavior than the individuals born to fathers in the middle of the paternal age distribution. There were no differences in development in other domains related to paternal age at conception.

Importantly, these results fit the epidemiological data linking APA with disorders associated with social deficits, and expand previous findings from animal models. In a study in mice, we previously showed that offspring of aged fathers shifted from increased to decreased sociability between early development and adulthood,^26^ patterns resembling those identified here. Such convergence of findings from animal and human studies is particularly encouraging, given the complementary strengths of these methodologies. Conclusions drawn from our animal study had to be taken with caution due to limitations in the experimental design (measuring sociability at two time points only, using different sets of inbred animals on both occasions). These problems could be addressed in the current study, where measures from up to five time points were considered within the same individuals. On the other hand, the murine study provided evidence that the differences in offspring most likely arose de novo (no differences in paternal behavior), as they occurred even when both familial genetic and environmental factors were strictly controlled. Obtaining similar results from these two studies reduces the likelihood that either murine or human results are just artifactual and can be explained solely by the limitations inherent in the methods.

The patterns seen in both species may suggest a degree of disinhibition or lack of selectivity in the early social approach of offspring born to aged fathers. Further studies exploring whether early increase in sociability occurs to a level that could be considered inappropriate, and ideally augmenting these results with molecular and neuroimaging data, will be necessary to fully understand the results of this study. However, it is interesting to note a parallel with findings from a study of infants at genetic risk of autism. Jones and Klin (2013)^49^ reported decreasing rates of looking at the eye region of faces from 2 to 6 months in those infants later diagnosed with autism. This abnormal trajectory reflected, in part, higher rates of looking at eyes at 2 months compared to those of typically developing infants.

### Heritable Differences

Our study sample offered a scope to investigate the genetic and environmental underpinnings of the observed developmental trajectories of social behavior, and to estimate how they change in relation to paternal age at conception.

We hypothesized that genetic variance in developmental measures would be moderated by paternal age only in offspring of very old fathers, and environmental variance only in offspring of very young fathers. Under this model, our results from the social trajectory analysis, although similar in these two groups, have distinct genetic and environmental underpinnings. These predictions are in line with previous research suggesting qualitative differences in paternal age effects between the two extremes of the paternal age distribution^50^ and exponential increase in de novo mutations in paternal spermatogonia.^15^ The results from the twin analysis stratified by paternal age supported this notion, indicating stable heritability estimates in the offspring of men <45 years old at conception, with a sharp increase in the offspring of older fathers. These trends, however, were not validated in the GxE analysis, likely because of insufficient statistical power in our sample to estimate such nonlinear effects.

An alternative explanation for the lack of significant results in the GxE analysis is that paternal age effects on social function arise mostly because of familial influences, that is, that there is a correlation between genetic factors influencing age at conception and social development. If paternal age at conception affects prosocial scores predominantly via such familial (non−de novo) genetic factors, regressing out the genetic effects on age at fatherhood would result in nonsignificant effects of paternal age on the genetic variance, as we recorded in the GxE model. Lack of significant effects of the GxE analysis, therefore, does not allow us to disentangle whether the increase in heritability in the offspring of the oldest fathers observed in the ACE model is more likely due to familial or to de novo factors. Although our earlier study in mice suggested that these former effects were unlikely (mice were inbred, and group differences recorded in offspring were not detected in their fathers), our current results did not provide support for this notion.

### Maternal Age Effects

Although maternal age effects were not of primary interest in our study, our results indicated that very young maternal age at conception is associated with a number of developmental differences. Offspring of young mothers showed increased levels of hyperactivity and conduct problems, as well as lower emotionality. This partly corroborates previous results showing association between young maternal age and externalizing problems.^51^, ^52^ Although the effects of young motherhood on offspring adversity can be explained partly by lower SES^53^ and obstetric complications,^54^ there is evidence suggesting possible association between contribution of familial genetic^55^ factors.

Although evidence suggests that the APA effects could be particularly strong in female offspring (higher disorder risk,^3^, ^6^ earlier onset and worse prognosis^56^), due to the small number of offspring with parents in the oldest categories, we could not investigate sex-specific trajectories. In our model, offspring sex was included as a covariate. Future studies should investigate whether females born to older men depart from their expected (typical) developmental patterns more than males.

Furthermore, although parental age categories used in this study were based on previous research (e.g., Lundström *et al.*, 2010),^36^ their determination was somewhat arbitrary. Given the complex and nondeterministic nature of the APA effects—with most offspring of aged parents experiencing no behavioral difficulties—the use of mixed-effects growth models in the future could help to identify more homogeneous (latent) subgroups. Subsequent analyses could determine whether these subgroups are enriched in offspring with fathers from any particular age range. Additional integration of biological data will help attribute these effects to factors such as total de novo burden, DNA methylation alterations in candidate regions, or neuroimaging results, allowing one to test new hypotheses about the biological underpinnings of the APA effects.

In conclusion, in this study paternal age specifically affected developmental trajectories of offspring social behaviors, as indexed by the prosocial subscale of the SDQ. Results of APA effects in the study sample were comparable to observations in recent animal models, with both suggesting developmental reversal from hyper- to hyposociability in the offspring of men in either the lower or upper extreme of paternal age.

## Figures and Tables

**Figure 1:**
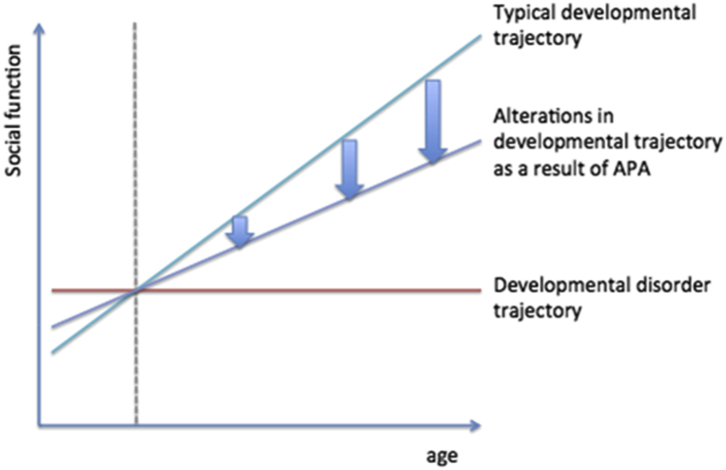
Illustration of putative effects of advanced paternal age (APA) on the trajectory of social development. Note: Although a considerable proportion of offspring of older fathers do not meet the criteria for clinical diagnosis of autism/schizophrenia (red line), their developmental profile is shifted from the one observed in typically developing individuals.

**Table 1:** Sample Characteristics by Paternal Age

Paternal Age Group	Median Age at Data Collection Wave					Mean Maternal Age	Mean SES	% Female	% MZ	n
	4	7	9	12	16					
PAC1	4.0	7.1	9.0	11.6	16.6	24.0 (4.4)	−0.9 (0.7)	47	47	1,270
PAC2	4.0	7.0	9.0	11.5	16.5	29.3 (3.8)	−0.1 (0.9)	51	35	13,382
PAC3	4.0	7.0	9.0	11.4	16.4	33.6 (3.8)	0.4 (1.0)	51	28	6,852
PAC4	4.0	7.0	9.1	11.4	16.5	34.5 (4.6)	0.4 (0.9)	55	28	649
PAC5	4.0	7.1	9.1	11.5	16.5	35.1 (4.3)	0.5 (1.0)	50	26	169

Note: Median age at each data collection wave, mean maternal age, mean socioeconomic status (SES; standardized, score reflects number of standard deviations from the mean) and percentage of females and monozygotic (MZ) twins are presented with their standard deviations, where relevant. PAC = paternal age category.

**Table 2:** Mean Strengths and Difficulties Questionnaire Prosocial Scores per Paternal Age Group in Each Data Collection Wave

	PAC1	PAC2	PAC3	PAC4	PAC5
4	7.58 (0.1)	7.41 (0.0)	7.31 (0.0)	7.24 (0.1)	7.86 (0.2)
7	8.32 (0.1)	8.24 (0.0)	8.14 (0.0)	8.17 (0.1)	8.33 (0.2)
9	8.40 (0.1)	8.31 (0.0)	8.29 (0.0)	8.04 (0.1)	8.20 (0.3)
12	8.64 (0.1)	8.58 (0.0)	8.58 (0.0)	8.34 (0.1)	8.55 (0.2)
16	8.24 (0.1)	8.30 (0.0)	8.23 (0.0)	7.95 (0.1)	7.82 (0.2)

Note: Standard errors are presented in parentheses. PAC = paternal age category.

**Table 3:** Standardized Genetic and Environmental Variance Components for the Parameters of the Strengths and Difficulties Questionnaire (SDQ) Growth Curves

Full Sample	Intercept	Slope
h^2^	0.68 (0.65–0.70)	0.71 (0.69–0.73)
e^2^	0.32 (0.30–0.35)	0.29 (0.27–0.31)

Stratified by Paternal Age	Paternal Age	Intercept	Slope
h^2^	>25	0.53 (0.08–0.70)	0.48 (0.07–0.73)
	25–34	0.58 (0.47–0.69)	0.52 (0.42–0.62)
	35–44	0.45 (0.29–0.61)	0.40 (0.25–0.55)
	>44	0.69 (0.36–0.80)	0.72 (0.39–0.82)
c^2^	>25	0.06 (0–0.43)	0.16 (0–0.49)
	25–34	0.12 (0.02–0.21)	0.21 (0.12–0.30)
	35–44	0.16 (0.03–0.28)	0.24 (0.11–0.36)
	>44	0 (0–0.25)	0 (0–0.26)
e^2^	>25	0.41 (0.30–0.5)	0.37 (0.26–0.51)
	25–34	0.30 (0.27–0.33)	0.27 (0.24–0.30)
	35–44	0.39 (0.34–0.46)	0.36 (0.31–0.42)
	>44	0.31 (0.20–0.47)	0.28 (0.18–0.43)

Note: h^2^, c^2^, and e^2^ represent standardized variance components derived from the Additive genetics, Common environment, unique Environment (ACE) model, representing proportion of variance explained by, in order, additive genetic (h^2^), common environmental (c^2^), and unique environmental (e^2^) effects. Estimates are given with their 95% CIs (in parentheses). Upper panel represents results from the entire sample, regardless of paternal age. Lower panel represents results stratified by paternal age. Shared environmental influences were non-zero only in the stratified analyses.
