# Corrigendum

**DOI:** 10.1016/j.jaac.2017.05.013

**Published:** 2017-07

In the funding information for open access to the research article “Paternal Age Alters Social Development in Offspring” by Janecka et al., published in the May 2017 issue of the *Journal of the American Academy of Child and Adolescent Psychiatry* (2017;56:383-390), the Medical Research Council was incorrectly credited as the sponsor. Open access was funded by the Seaver Autism Center for Research and Treatment and the Icahn School of Medicine at Mount Sinai.

