# Supplementary material for: Paternal Age Alters Social Development in Offspring
Source: J Am Acad Child Adolesc Psychiatry. 2017 May;56(5):383–90. doi: 10.1016/j.jaac.2017.02.006 (PMC5409803; doi:10.1016/j.jaac.2017.02.006)
Supplement: Supplemental Material [file mmc1.docx]

SUPPLEMENTARY MATERIAL

METHOD

### Multilevel (Mixed) Models for Longitudinal Data

Using multilevel modelling allowed us to derive the intercept and the slope coefficients, which describe developmental change in the prosocial scores. An intercept reflects the mean group value at the starting point, and the slope reflects the developmental growth. Higher initial sociability was indicated by high values of the prosocial intercept, and increase in sociability over time by its positive slope. Significant effects of paternal age on the slope coefficients would suggest social development is influenced by father’s age at conception. We hypothesized no group differences in the intercept coefficient, reflecting the prediction that offspring of older fathers would not display striking differences in sociability very early in life.

To account for the clustering of observations both within individuals (given the longitudinal design) and families (given the twin-pair structure of the data), both of these structures were entered into the model as random effects on the intercept (R package *lme4*). Actual age at completing the questionnaires rather than the data collection wave was used as the time predictor, thus widening the age range of the participants. We investigated the effects of paternal age as a categorical variable, running a series of pairwise comparisons between the paternal age category (PAC)-dependent coefficients to determine significant group differences. Furthermore, to exploit the full range of paternal ages in the sample, we ran trend analyses, with paternal age as a continuous predictor of the intercept and slope coefficients. Both approaches are described in detail below.

#### Pairwise Comparisons

Both intercept and slope of the curve for each paternal age category were estimated in a single linear model, involving a series of dummy variables corresponding to different paternal age groups (PAC1-5, yes/no). In the fully adjusted model, paternal and maternal ages, sex, zygosity, and socioeconomic status (SES, index of parent qualifications and employment, and mother’s age at birth of first child) were entered as fixed effects (without interaction terms). The shared intercept term was removed from the regression to facilitate interpretation of paternal age-specific intercepts. To determine if the differences between the coefficients were significantly different from each other, a series of pairwise comparisons were performed simultaneously (separately for the slope and intercept in each of the tests) using the R package *multcomp*. We adjusted for multiple testing with the false discovery rate method,^1^ and all presented p-values represent post-adjustment scores. Assuming missing-at-random, all individuals, even ones contributing fewer than the maximum number of time-point observations, were included in the analysis. To obtain smooth curves based on the results of the regression, the locally weighted scatterplot smoothing method was used. Figures in the Results section present model predictions of social development trajectories in relation to paternal age category. Participants’ ages presented on the y-axis go beyond the latest data collection wave (age 16) due to a 2-5 years range of participants’ ages at each data collection wave. Quadratic and higher-order time effects were not considered in any of the models, due to absence of a strong hypothesis about why such effects would come into play.

#### Developmental Analysis

To examine the effect of paternal age effects on the growth curve coefficients, prosocial slope and intercept scores were extracted for each individual after the growth curve model analysis, using the crude model (paternal age effects only). Multilevel models were run as described above, except that only the family ID was entered as a random effect, as each individual contributed only a single point to each analysis. To select the best-fitting model, quadratic and linear models, estimated using maximum likelihood procedure, were compared using a Chi^2^ test. Paternal age effects on the intercept and slope scores were estimated in both crude and adjusted models. In this set of analyses, both paternal and maternal ages were entered into the model as continuous variables.

### Twin Model-Fitting Analysis

In the second stage, we investigated the extent to which individual differences in derived trajectory parameter scores could be explained by latent genetic and environmental effects, as well as how these effects change with paternal age. Our approach allowed us to elucidate whether the effects of paternal age on the heritability estimates operate in a linear vs. nonlinear fashion, as well as their de novo vs. inherited nature.

Our prediction was that both environmental and genetic variance would change in a nonlinear fashion across paternal age range, reflecting different aetiology of the effects in very young and very old fathers. We hypothesized that the environmental variance would be predominantly affected in offspring of very young fathers, with little variation in those born to middle-aged to old men. Conversely, our prediction was that genetic variance would be moderated by paternal age only in the oldest fathers, due to age-related genetic changes exerting their effect only past a certain threshold.

#### Additive Genetics, Common Environment, Unique Environment (ACE) Analysis

All analyses were run in both the full sample and stratified by paternal age (PAC 1-4 categories; PAC4 and PAC5 groups were collapsed for these analyses due to insufficient number of individuals with very old fathers to perform twin analyses). While the former informs about the degree to which social development parameters are heritable in the population, the latter approach allowed us to observe whether the heritability estimates are stable across different paternal age groups. For all analyses, we used the individual slope and intercept scores, derived using crude models. Given that heritability estimates may vary with age, with studies in younger participants often reporting higher proportions of shared environmental influences, we truncated the range of prosocial scores used for these analyses, using only data from years 7, 9, and 12 (year 7 rather than 9 was chosen as the lower boundary due to the latter contributing substantially fewer data points, affecting estimates of the intercept). Tetrachoric correlations for these scores in mono- (MZ) and dizygotic (DZ) twins were derived using full information maximum likelihood estimation. To obtain standardized genetic and environmental variance components, we ran ACE and AE models for the SDQ coefficients. For more details on these models, see ^2^ or ^3^.

#### Gene–Environment (GxE) Analysis

To gain further insights into the potential moderating effects of paternal age on the genetic and environmental variance components, as well as the de novo vs. inherited nature of the former, we also ran a nonlinear GxE model. This allowed us to partition the genetic and environmental effects on the coefficients of the growth curves into those that are moderated and those that are independent of paternal age. To limit the possibility that the moderating effects on the variance components in these models are biased by a genetic correlation between moderator (paternal age) and growth curve coefficients, paternal age effects were regressed out of the slope and intercept coefficients prior to fitting the GxE model. For all individuals with missing data on paternal age, the variable was imputed at the sample mean (33.37 years).

In order to retain focus on the paternal age effects and to avoid multiple testing, twin modelling on the slope and intercept scores was performed in relation to paternal but not maternal age. Taking a developmental perspective, we predicted that these genetic effects would be observed mainly on the derived slope variable, either with or without concurrent effects on the intercept. In line with our hypothesis that the genetic effects acting on social development are affected by paternal age at conception in a nonlinear manner, we predicted that (i) in the stratified ACE/AE analyses, the differences in the heritability estimates would be most pronounced between the offspring of oldest fathers and other groups, and (ii) in the GxE analyses, the quadratic coefficient of moderating effects of paternal age on genetic variance would be significant.

**Supplemental References**

1. Gur RE, Nimgaonkar VL, Almasy L, et al. Neurocognitive endophenotypes in a multiplex multigenerational family study of schizophrenia. *Am J Psychiatry*. 2007;164:813-819.

2. Plomin R, Haworth CM, Davis OSP. Common disorders are quantitative traits. *Nat Rev Genet*. 2009;10:872-8.

3. Happé F, Ronald A, Plomin R. Time to give up on a single explanation for autism. *Nat Neurosci*. 2006;9:1218-20.

SUPPLEMENTARY TABLES

| Table S1. Linear and Quadratic (Where Justified by Model Fit Indices) Effects of Paternal Age on Strengths and Difficulties Questionnaire Growth Curve Coefficients, Crude Model, Sample After Exclusions | | | |
| --- | --- | --- | --- |
|  |  | **paternal age** | **paternal age^2^** |
| Prosocial | slope | β=9.40E^-3^, p=.03 | β=-1.35E^-4^, p=.03 |
|  | intercept | β=-0.13, p=9.56E^-4^ | β=1.66E^-3^, p=1.93E^-3^ |
| Peer problems | slope | β=-1.17E^-3^, p=.07 | - |
|  | intercept | β=7.70E^-3^, p=.13 | - |
| Hyperactivity | slope | β<0.01, p=.15 | - |
|  | intercept | β=-0.20, p=6.64E^-5^ | β=2.38E^-3^, p=7.00E^-4^ |
| Emotionality | slope | β=-0.017, p=4.70E^-4^ | - |
|  | intercept | β=9.21E^-4^, p=.145 | - |
| Conduct problems | slope | β=5.66E^-4^, p=.35 | - |
|  | intercept | β=-0.12, p=1.93E-3 | β=1.41E-3, p=7.56E-3 |

| Table S2. Linear and Quadratic (Where Justified by Model Fit Indices) Effects of Paternal Age on Strengths and Difficulties Questionnaire Growth Curve Coefficients, Adjusted Model, Sample After Exclusions | | | | |
| --- | --- | --- | --- | --- |
|  |  | **paternal age** | **paternal age^2^** | **maternal age** |
| Prosocial | slope | β=9.72E^-3^, p=.03 | β=1.39E^-4^, p=.03 | β=3.19E^-4^, p=.76 |
|  | intercept | β=-0.10, p=.02 | β=1.37E^-3^, p=.01 | β=-0.01, p=.41 |
| Peer problems | slope | β=7.87E-4, p=.33 | - | β=-2.02E^-4^, p=.86 |
|  | intercept | β=-0.02, p=.64 | - | β=0.01, p=.34 |
| Hyperactivity | slope | β<0.01, p=.87 | - | β<0.01, p=.13 |
|  | intercept | β=-0.09, p=.10 | β<0.01, p=.12 | β<0.01, p=.67 |
| Emotionality | slope | β=2.92E^-4^, p=.71 | - | β=2.0E-3, p=.09 |
|  | intercept | β=-6.0E-3, p=.30 | - | β=-0.01, p=.11 |
| Conduct problems | slope | β=4.04E^-5^, p=.96 | - | β=1.49E^-4^, p=.89 |
|  | intercept | β=-0.05, p=.22 | β<0.01, p=.23 | β=0.01, p=.44 |

| Table S3. Linear and Quadratic Effects of Paternal Age on Strengths and Difficulties Questionnaire (SDQ) Slope and Intercept Coefficients, Crude Model, Sample Without Exclusions | | | | |
| --- | --- | --- | --- | --- |
|  | **crude model** | | **adjusted model** | |
|  | **paternal age** | **paternal age^2^** | **paternal age** | **paternal age^2^** |
| SDQ slope | β=7.80E^-3^, p=.07 | β=-1.11E^-4^, p=.07 | β=8.34E^-3^, p=.07 | β=-1.20E^-4^, p=.05 |
| SDQ intercept | β= -0.11, p= 2.09E^-3^ | β=1.50E^-3^, p=4.20E^-3^ | β=-0.09^-3^, p=.03 | β=0.001, p=.02 |

| Table S4. Coefficients and Their Pairwise Comparisons for the Strengths and Difficulties Questionnaire (SDQ) Prosocial Trajectory Unadjusted Model (Paternal Age Categories [PAC] Only), Sample After Exclusions | | | | | |
| --- | --- | --- | --- | --- | --- |
| **SDQ PROSOCIAL TRAJECTORY – sample after exclusions** | | | | | |
| **UNADJUSTED MODEL (PAC)** | | | | | |
| **INTERCEPT (PAC)** | | | | | |
|  | PAC1 | PAC2 | PAC3 | PAC4 | PAC5 |
| PAC1 | 7.68 (0.09) | . | . | . | . |
| PAC2 | 0.24 (0.09) | 7.44 (0.03) | . | . | . |
| PAC3 | 0.32 (0.10) | 0.08 (0.04) | 7.36 (0.03) | . | . |
| PAC4 | 0.29 (0.14) | 0.05 (0.11) | -0.03 (0.12) | 7.39 (0.11) | . |
| PAC5 | -0.40 (0.24) | -0.64 (0.22) | -0.72 (0.22) | -0.69 (0.25) | 8.08 (0.22) |
|  | | | | | |
| **SLOPE (PAC)** | | | | | |
|  | PAC1 | PAC2 | PAC3 | PAC4 | PAC5 |
|  | 0.05 (0.01) | . | . | . | . |
| PAC2 | -0.02 (0.01)* | 0.07 (0.00) | . | . | . |
| PAC3 | -0.03 (0.01)** | -0.00 (0.00) | 0.08 (0.00) | . | . |
| PAC4 | -0.00 (0.01) | 0.02 (0.01) | 0.02 (0.01) | 0.05 (0.01) | . |
| PAC5 | 0.04 (0.02) | 0.06 (0.02)** | 0.06 (0.02)** | 0.04 (0.02) | 0.01 (0.02) |
| n | 855 | 9,835 | 5,443 | 492 | 121 |

Note. Intercept and slope values for each PAC group are presented on the diagonal. Pairwise comparisons between respective groups are in the remaining cells. Standard error of the estimates is shown in parentheses.

| Table S5. Coefficients and Their Pairwise Comparisons for the Strengths and Difficulties Questionnaire (SDQ) Peer Problems Trajectory Unadjusted Model (Paternal Age Categories [PAC] Only), Sample After Exclusions | | | | | |
| --- | --- | --- | --- | --- | --- |
| **SDQ PEER PROBLEMS TRAJECTORY – sample after exclusions** | | | | | |
| **UNADJUSTED MODEL (PAC)** | | | | | |
| **INTERCEPT (PAC)** | | | | | |
|  | PAC1 | PAC2 | PAC3 | PAC4 | PAC5 |
| PAC1 | 1.58 (0.09) | . | . | . | . |
| PAC2 | 0.05 (0.09) | 1.52 (0.02) | . | . | . |
| PAC3 | 0.03 (0.09) | -0.02 (0.04) | 1.55 (0.03) | . | . |
| PAC4 | 0.05 (0.14) | <0.01 (0.11) | 0.03 (0.11) | 1.52 (0.11) | . |
| PAC5 | -0.09 (0.24) | -0.14 (0.22) | -0.12 (0.22) | -0.15 (0.24) | 1.67 (0.22) |
|  | | | | | |
| **SLOPE (PAC)** | | | | | |
|  | PAC1 | PAC2 | PAC3 | PAC4 | PAC5 |
| PAC1 | -0.03 (0.01) | . | . | . | . |
| PAC2 | 0.02 (0.01) | -0.05 (<0.01) | . | . | . |
| PAC3 | 0.03 (0.01) | 0.01 (<0.01) | -0.06 (<0.01) | . | . |
| PAC4 | 0.01 (0.02) | -0.02 (0.01) | -0.02 (0.01) | -0.04 (0.01) | . |
| PAC5 | 0.02 (0.03) | -0.01 (0.02) | -0.01 (0.02) | 0.01 (0.03) | -0.05 (0.02) |
| n | 855 | 9,835 | 5,443 | 492 | 121 |

Note. Intercept and slope values for each PAC group are presented on the diagonal. Pairwise comparisons between respective groups are in the remaining cells. Standard error of the estimates is shown in parentheses.

| Table S6. Coefficients and Their Pairwise Comparisons for the Strengths and Difficulties Questionnaire (SDQ) Hyperactivity Trajectory Unadjusted Model (Paternal Age Categories [PAC] Only), Sample After Exclusions | | | | | |
| --- | --- | --- | --- | --- | --- |
| **SDQ HYPERACTIVITY TRAJECTORY – sample after exclusions** | | | | | |
| **UNADJUSTED MODEL (PAC)** | | | | | |
| **INTERCEPT (PAC)** | | | | | |
|  | PAC1 | PAC2 | PAC3 | PAC4 | PAC5 |
| PAC1 | 5.08 (0.13) | . | . | . | . |
| PAC2 | 0.34 (0.13) | 4.73 (0.04) | . | . | . |
| PAC3 | 0.66 (0.14)** | 0.32 (0.06)** | 4.42 (0.05) | . | . |
| PAC4 | 0.85 (0.20)** | 0.50 (0.16)* | 0.18 (0.16) | 4.23 (0.16) | . |
| PAC5 | 0.31 (0.06) | -0.03 (0.32) | -0.35 (0.32) | -0.53 (0.35) | 4.76 (0.32) |
|  | | | | | |
| **SLOPE (PAC)** | | | | | |
|  | PAC1 | PAC2 | PAC3 | PAC4 | PAC5 |
| PAC1 | -0.16 (0.01) | . | . | . | . |
| PAC2 | <0.01 (0.01) | -0.16 (<0.01) | . | . | . |
| PAC3 | <-0.01 (0.01) | -0.01 (0.01) | -0.15 (<0.01) | . | . |
| PAC4 | -0.02 (0.02) | -0.02 (0.02) | -0.01 (0.02) | -0.14 (0.02) | . |
| PAC5 | 0.02 (0.04) | 0.02 (0.03) | 0.03 (0.03) | 0.04 (0.04) | -0.18 (0.03) |
| n | 855 | 9,835 | 5,443 | 492 | 121 |

Note. Intercept and slope values for each PAC group are presented on the diagonal. Pairwise comparisons between respective groups are in the remaining cells. Standard error of the estimates is shown in parentheses.

| Table S7. Coefficients and Their Pairwise Comparisons for the Strengths and Difficulties Questionnaire (SDQ) Emotionality Trajectory Unadjusted Model (Paternal Age Categories [PAC] Only), Sample After Exclusions | | | | | |
| --- | --- | --- | --- | --- | --- |
| **SDQ EMOTIONALITY TRAJECTORY – sample after exclusions** | | | | | |
| **UNADJUSTED MODEL (PAC)** | | | | | |
| **INTERCEPT (PAC)** | | | | | |
|  | PAC1 | PAC2 | PAC3 | PAC4 | PAC5 |
| PAC1 | 1.89 (0.09) | . | . | . | . |
| PAC2 | 0.33 (0.09)** | 1.56 (0.02) | . | . | . |
| PAC3 | 0.42 (0.09)** | 0.09 (0.04) | 1.47 (0.03) | . | . |
| PAC4 | 0.71 (0.14)** | 0.38 (0.11) | 0.29 (0.12) | 1.18 (0.11) | . |
| PAC5 | 0.29 (0.24) | -0.04 (0.22) | -0.13 (0.22) | -0.42 (0.25) | 1.60 (0.22) |
|  | | | | | |
| **SLOPE (PAC)** | | | | | |
|  | PAC1 | PAC2 | PAC3 | PAC4 | PAC5 |
| PAC1 | -0.03 (0.01) |  |  |  |  |
| PAC2 | -0.01 (0.01) | -0.02 (<0.01) |  |  |  |
| PAC3 | -0.02 (0.01) | <-0.01 (<0.01) | -0.01 (<0.01) |  |  |
| PAC4 | -0.05 (0.02)* | -0.04 (<0.01)* | -0.03 (0.01) | 0.02 (0.01) |  |
| PAC5 | <-0.01 (0.03) | 0.01 (0.02) | 0.01 (0.02) | 0.05 (0.03) | -0.03 (0.02) |
| n | 855 | 9,835 | 5,443 | 492 | 121 |

Note. Intercept and slope values for each PAC group are presented on the diagonal. Pairwise comparisons between respective groups are in the remaining cells. Standard error of the estimates is shown in parentheses.

| Table S8. Coefficients and Their Pairwise Comparisons for the Strengths and Difficulties Questionnaire (SDQ) Conduct Problems Trajectory Unadjusted Model (Paternal Age Categories [PAC] Only), Sample After Exclusions | | | | | |
| --- | --- | --- | --- | --- | --- |
| **SDQ CONDUCT PROBLEMS TRAJECTORY – sample after exclusions** | | | | | |
| **UNADJUSTED MODEL (PAC)** | | | | | |
| **INTERCEPT (PAC)** | | | | | |
|  | PAC1 | PAC2 | PAC3 | PAC4 | PAC5 |
| PAC1 | 2.86 (0.09) | . | . | . | . |
| PAC2 | 0.39 (0.09)** | 2.47 (0.03) | . | . | . |
| PAC3 | 0.049 (0.10)** | 0.10 (0.04) | 2.37 (0.03) | . | . |
| PAC4 | 0.74 (0.14)** | 0.35 (0.11)* | 0.25 (0.12) | 2.12 (0.11) | . |
| PAC5 | 0.50 (0.24) | 0.10 (0.22) | <0.01 (0.22) | -0.25 (0.25) | 2.37 (0.22) |
|  | | | | | |
| **SLOPE (PAC)** | | | | | |
|  | PAC1 | PAC2 | PAC3 | PAC4 | PAC5 |
| PAC1 | -0.10 (0.01) | . | . | . | . |
| PAC2 | <0.01 (0.01) | -0.11 (<0.01) | . | . | . |
| PAC3 | <0.01 (0.01) | <0.01 (<0.01) | -0.11 (<0.01) | . | . |
| PAC4 | -0.02 (0.02) | -0.02 (0.01) | -0.03 (0.01) | -0.08 (0.01) | . |
| PAC5 | -0.02 (0.03) | -0.02 (0.02) | -0.03 (0.02) | <0.01 (0.03) | -0.08 (0.02) |
| n | 855 | 9835 | 5443 | 492 | 121 |

Note. Intercept and slope values for each PAC group are presented on the diagonal. Pairwise comparisons between respective groups are in the remaining cells. Standard error of the estimates is shown in parentheses.

| Table S9. Coefficients and Their Pairwise Comparisons for the Strengths and Difficulties Questionnaire (SDQ) Prosocial Trajectory in a Fully Adjusted Model (Paternal [PAC] and Maternal [MAC] Age Categories, Offspring Sex and Zygosity and Family’s Socioeconomic Status [SES]) | | | | | |
| --- | --- | --- | --- | --- | --- |
| **SDQ PROSOCIAL TRAJECTORY – sample after exclusions** | | | | | |
| **ADJUSTED MODEL (SEX, SES, MATERNAL AGE, ZYGOSITY)** | | | | | |
| **SDQ INTERCEPT - PAC** | | | | | |
|  | PAC1 | PAC2 | PAC3 | PAC4 | PAC5 |
| PAC1 | 4.96 (1.96) | . | . | . | . |
| PAC2 | 0.26 (0.10) | 4.70 (1.95) | . | . | . |
| PAC3 | 0.31 (0.11)* | 0.05 (0.05) | 4.65 (1.95) | . | . |
| PAC4 | 0.13 (0.16) | -0.13 (0.12) | -0.18 (0.12) | 4.83 (1.96) | . |
| PAC5 | -0.42 (0.25) | -0.68 (0.23)* | -0.73 (0.23)** | -0.55 (0.25) | 5.38 (1.97) |
|  | | | | | |
| **SDQ SLOPE - PAC** | | | | | |
|  | PAC1 | PAC2 | PAC3 | PAC4 | PAC5 |
| PAC1 | 0.51 (0.21) | . | . | . | . |
| PAC2 | -0.02 (0.01) | 0.53 (0.21) | . | . | . |
| PAC3 | -0.03 (0.01)* | -0.01 (0.00) | 0.54 (0.21) | . | . |
| PAC4 | 0.01 (0.01) | 0.03 (0.02)* | 0.03 (0.01)** | 0.50 (0.21) | . |
| PAC5 | 0.03 (0.02) | 0.05 (0.02)* | 0.06 (0.02)** | 0.03 (0.02) | 0.48 (0.21) |
| n | 735 | 9,388 | 5,167 | 462 | 113 |

Note. Intercept and slope values for each PAC/MAC group are presented on the diagonal. Pairwise comparisons between respective groups are in the remaining cells. Standard error of the estimates is shown in parentheses. * Denotes tests significant at *p*=.05, and ** at *p*=.01.

| Table S10. Coefficients and Their Pairwise Comparisons for the Strengths and Difficulties Questionnaire (SDQ) Peer Problems Trajectory in a Fully Adjusted Model (Paternal [PAC] and Maternal [MAC] Age Categories, Offspring Sex and Zygosity and Family’s Socioeconomic Status [SES]) | | | | | |
| --- | --- | --- | --- | --- | --- |
| **SDQ PEER PROBLEMS TRAJECTORY – sample after exclusions** | | | | | |
| **ADJUSTED MODEL (SEX, SES, MATERNAL AGE, ZYGOSITY)** | | | | | |
| **SDQ INTERCEPT - PAC** | | | | | |
|  | PAC1 | PAC2 | PAC3 | PAC4 | PAC5 |
| PAC1 | 0.61 (1.57) | . | . | . | . |
| PAC2 | -0.05 (0.10) | 0.67 (1.57) | . | . | . |
| PAC3 | -0.08 (0.11) | -0.03 (0.05) | 0.70 (1.57) | . | . |
| PAC4 | <-0.01 (0.15) | 0.05 (0.12) | 0.08 (0.12) | 0.62 (1.57) | . |
| PAC5 | -0.13 (0.25) | -0.08 (0.23) | -0.05 (0.22) | -0.12 (0.25) | 0.74 (1.58) |
|  | | | | | |
| **SDQ SLOPE - PAC** | | | | | |
|  | PAC1 | PAC2 | PAC3 | PAC4 | PAC5 |
| PAC1 | -0.05 (0.17) | . | . | . | . |
| PAC2 | 0.02 (0.01) | -0.06 (0.17) | . | . | . |
| PAC3 | 0.02 (0.01) | <0.01 (0.01) | -0.07 (0.17) | . | . |
| PAC4 | <-0.01 (0.02) | -0.02 (0.01) | -0.02 (0.01) | -0.04 (0.17) | . |
| PAC5 | <0.01 (0.02) | -0.02 (0.03) | -0.02 (0.03) | <0.01 (0.03) | -0.05 (0.18) |
| n | 735 | 9,388 | 5,167 | 462 | 113 |

Note. Intercept and slope values for each PAC/MAC group are presented on the diagonal. Pairwise comparisons between respective groups are in the remaining cells. Standard error of the estimates is shown in parentheses. * Denotes tests significant at *p*=.05, and ** at *p*=.01.

| Table S11. Coefficients and Their Pairwise Comparisons for the Strengths and Difficulties Questionnaire (SDQ) Hyperactivity Trajectory in a Fully Adjusted Model (Paternal [PAC] and Maternal [MAC] Age Categories, Offspring Sex and Zygosity and Family’s Socioeconomic Status [SES]) | | | | | |
| --- | --- | --- | --- | --- | --- |
| **SDQ HYPERACTIVITY TRAJECTORY – sample after exclusions** | | | | | |
| **ADJUSTED MODEL (SEX, SES, MATERNAL AGE, ZYGOSITY)** | | | | | |
| **SDQ INTERCEPT - PAC** | | | | | |
|  | PAC1 | PAC2 | PAC3 | PAC4 | PAC5 |
| PAC1 | 4.06 (2.26) | . | . | . | . |
| PAC2 | -0.27 (0.14) | 4.33 (2.25) | . | . | . |
| PAC3 | -0.16 (0.15) | 0.12 (0.06) | 4.22 (2.25) | . | . |
| PAC4 | 0.02 (0.21) | 0.29 (0.16) | 0.18 (0.16) | 4.04 (2.26) | . |
| PAC5 | -0.45 (0.35) | -0.17 (0.32) | -0.29 (0.32) | -0.47 (0.35) | 4.51 (2.27) |
|  | | | | | |
| **SDQ SLOPE - PAC** | | | | | |
|  | PAC1 | PAC2 | PAC3 | PAC4 | PAC5 |
| PAC1 | -0.09 (0.23) | . | . | . | . |
| PAC2 | 0.03 (0.02) | -0.13 (0.23) | . | . | . |
| PAC3 | 0.03 (0.02) | <-0.01 (0.01) | -0.12 (0.23) | . | . |
| PAC4 | 0.01 (0.02) | -0.02 (0.02) | -0.02 (0.02) | -0.11 (0.23) | . |
| PAC5 | 0.04 (0.04) | 0.01 (0.03) | 0.01 (0.03) | 0.03 (0.04) | -0.14 (0.24) |
| n | 735 | 9,388 | 5,167 | 462 | 113 |

Note. Intercept and slope values for each PAC group are presented on the diagonal. Pairwise comparisons between respective groups are in the remaining cells. Standard error of the estimates is shown in parentheses. * Denotes tests significant at *p*=.05, and ** at *p*=.01.

| S12. Coefficients and Their Pairwise Comparisons for the Strengths and Difficulties Questionnaire (SDQ) Hyperactivity Trajectory in a Fully Adjusted Model (Paternal [PAC] and Maternal [MAC] Age Categories, Offspring Sex and Zygosity and Family’s Socioeconomic Status [SES]) | | | | |
| --- | --- | --- | --- | --- |
| **SDQ HYPERACTIVITY TRAJECTORY – sample after exclusions** | | | | |
| **ADJUSTED MODEL (SEX, SES, MATERNAL AGE, ZYGOSITY)** | | | | |
| **SDQ INTERCEPT - PAC** | | | | |
|  | MAC1 | MAC2 | MAC3 | MAC4 |
| MAC1 | 0.43 (2.25) | . | . | . |
| MAC2 | 0.56 (0.11)** | -0.13 (2.25) | . | . |
| MAC3 | 0.47 (0.13)** | -0.08 (0.08) | -0.04 (2.25) | . |
| MAC4 | 0.76 (0.78) | 0.21 (0.86) | 0.29 (0.86) | -0.33 (2.41) |
|  | | | | |
| **SDQ SLOPE - PAC** | | | | |
|  | MAC1 | MAC2 | MAC3 | MAC4 |
| MAC1 | -0.09 (0.23) | . | . | . |
| MAC2 | -0.06 (0.01)** | -0.03 (0.23) | . | . |
| MAC3 | -0.07 (0.01)** | -0.01 (0.01) | -0.02 (0.23) | . |
| MAC4 | -0.04 (0.09) | 0.02 (0.09) | 0.02 (0.09) | -0.05 (0.25) |

Note. Intercept and slope values for each MAC group are presented on the diagonal. Pairwise comparisons between respective groups are in the remaining cells. Standard error of the estimates is shown in parentheses. * Denotes tests significant at *p*=.05, and ** at *p*=.01.

| Table S13. Coefficients and Their Pairwise Comparisons for the Strengths and Difficulties Questionnaire (SDQ) Emotionality Trajectory in a Fully Adjusted Model (Paternal [PAC] and Maternal [MAC] Age Categories, Offspring Sex and Zygosity and Family’s Socioeconomic Status [SES]) | | | | | |
| --- | --- | --- | --- | --- | --- |
| **SDQ EMOTIONALITY TRAJECTORY – sample after exclusions** | | | | | |
| **ADJUSTED MODEL (SEX, SES, MATERNAL AGE, ZYGOSITY)** | | | | | |
| **SDQ INTERCEPT - PAC** | | | | | |
|  | PAC1 | PAC2 | PAC3 | PAC4 | PAC5 |
| PAC1 | 2.06 (1.58) | . | . | . | . |
| PAC2 | 0.04 (0.10) | 2.02 (1.58) | . | . | . |
| PAC3 | 0.04 (0.11) | <0.01 (0.05) | 2.02 (1.58) | . | . |
| PAC4 | 0.37 (0.15) | 0.33 (0.12)* | 0.32 (0.12)* | 1.69 (1.58) | . |
| PAC5 | -0.08 (0.25) | -0.12 (0.23) | -0.13 (0.23) | -0.45 (0.25) | 2.14 (1.60) |
|  | | | | | |
| **SDQ SLOPE - PAC** | | | | | |
|  | PAC1 | PAC2 | PAC3 | PAC4 | PAC5 |
| PAC1 | -0.15 (0.17) |  |  |  |  |
| PAC2 | <0.01 (0.01) | -0.15 (0.17) |  |  |  |
| PAC3 | 0.01 (0.01) | <0.01 (0.01) | -0.15 (0.17) |  |  |
| PAC4 | -0.03 (0.02) | -0.03 (0.01) | -0.04 (0.01)* | -0.12 (0.17) |  |
| PAC5 | 0.02 (0.03) | 0.01 (0.03) | 0.01 (0.03) | 0.04 (0.03) | -0.16 (0.17) |
| n | 735 | 9388 | 5167 | 462 | 113 |

Note. Intercept and slope values for each PAC group are presented on the diagonal. Pairwise comparisons between respective groups are in the remaining cells. Standard error of the estimates is shown in parentheses. * Denotes tests significant at *p*=.05, and ** at *p*=.01.

| Table S14. Coefficients and Their Pairwise Comparisons for the Strengths and Difficulties Questionnaire (SDQ) Emotionality Trajectory in a Fully Adjusted Model (Paternal [PAC] and Maternal [MAC] Age Categories, Offspring Sex, and Zygosity and Family’s Socioeconomic Status [SES]) | | | | |
| --- | --- | --- | --- | --- |
| **SDQ EMOTIONALITY TRAJECTORY – sample after exclusions** | | | | |
| **ADJUSTED MODEL (SEX, SES, MATERNAL AGE, ZYGOSITY)** | | | | |
| **SDQ INTERCEPT - MAC** | | | | |
|  | MAC1 | MAC2 | MAC3 | MAC4 |
| MAC1 | -0.11 (1.58) | . | . | . |
| MAC2 | 0.38 (0.08)** | -0.49 (1.58) | . | . |
| MAC3 | 0.40 (0.09)** | 0.03 (0.05) | -0.52 (1.58) | . |
| MAC4 | 0.55 (0.61) | 0.17 (0.61) | -0.18 (0.61) | -0.66 (1.69) |
|  | | | | |
| **SDQ SLOPE - MAC** | | | | |
|  | MAC1 | MAC2 | MAC3 | MAC4 |
| MAC1 | 0.10 (0.17) | . | . | . |
| MAC2 | -0.03 (0.01)** | 0.14 (0.17) | . | . |
| MAC3 | -0.04 (0.01)** | -0.01 (0.01) | 0.15 (0.17) | . |
| MAC4 | <-0.01 (0.07) | 0.03 (0.07) | 0.04 (0.07) | 0.11 (0.19) |

Note. Intercept and slope values for each MAC group are presented on the diagonal. Pairwise comparisons between respective groups are in the remaining cells. Standard error of the estimates is shown in parentheses. * Denotes tests significant at *p*=.05, and ** at *p*=.01.

| Table S15. Coefficients and Their Pairwise Comparisons for the Strengths and Difficulties Questionnaire (SDQ) Conduct Problems Trajectory in a Fully Adjusted Model (Paternal [PAC] and Maternal [MAC] Age Categories, Offspring Sex and Zygosity and Family’s Socioeconomic Status [SES]) | | | | | |
| --- | --- | --- | --- | --- | --- |
| **SDQ CONDUCT PROBLEMS TRAJECTORY – sample after exclusions** | | | | | |
| **ADJUSTED MODEL (SEX, SES, MATERNAL AGE, ZYGOSITY)** | | | | | |
| **SDQ INTERCEPT - PAC** | | | | | |
|  | PAC1 | PAC2 | PAC3 | PAC4 | PAC5 |
| PAC1 | 2.47 (1.60) | . | . | . | . |
| PAC2 | 0.05 (0.10) | 2.42 (1.60) | . | . | . |
| PAC3 | 0.04 (0.11) | -0.01(0.05) | 2.43 (1.60) | . | . |
| PAC4 | 0.33 (0.15) | 0.28 (0.12) | 0.29 (0.12) | 2.14 (1.60) | . |
| PAC5 | -0.04 (0.25) | -0.08 (0.23) | -0.08 (0.23) | -0.37 (0.25) | 2.50 (1.61) |
|  | | | | | |
| **SDQ SLOPE - PAC** | | | | | |
|  | PAC1 | PAC2 | PAC3 | PAC4 | PAC5 |
| PAC1 | -0.06 (0.16) | . | . | . | . |
| PAC2 | <0.01 (0.01) | -0.07 (0.16) | . | . | . |
| PAC3 | 0.01 (0.01) | <0.01 (0.01) | -0.08 (0.16) | . | . |
| PAC4 | -0.03 (0.02) | -0.03 (0.01) | -0.04 (0.01) | -0.05 (0.16) | . |
| PAC5 | 0.02 (0.03) | 0.01 (0.03) | 0.01 (0.03) | 0.04 (0.03) | 0.05 (0.16) |
| n | 735 | 9388 | 5167 | 462 | 113 |

Note. Intercept and slope values for each PAC group are presented on the diagonal. Pairwise comparisons between respective groups are in the remaining cells. Standard error of the estimates is shown in parentheses. * Denotes tests significant at *p*=.05, and ** at *p*=.01.

| Table S16. Coefficients and Their Pairwise Comparisons for the Strengths and Difficulties Questionnaire (SDQ) Conduct Problems Trajectory in a Fully Adjusted Model (Paternal [PAC] and Maternal [MAC] Age Categories, Offspring Sex and Zygosity and Family’s Socioeconomic Status (SES) | | | | |
| --- | --- | --- | --- | --- |
| **SDQ CONDUCT PROBLEMS TRAJECTORY – sample after exclusions** | | | | |
| **ADJUSTED MODEL (SEX, SES, MATERNAL AGE, ZYGOSITY)** | | | | |
| **SDQ INTERCEPT - PAC** | | | | |
|  | MAC1 | MAC2 | MAC3 | MAC4 |
| MAC1 | 0.07 (1.60)** | . | . | . |
| MAC2 | 0.28 (0.08) | -0.21 (1.60) | . | . |
| MAC3 | 0.20 (0.09) | -0.08 (0.05) | -0.13 (1.60) | . |
| MAC4 | 0.09 (0.61) | -0.19 (0.60) | -0.11 (0.60) | -0.02 (1.71) |
|  | | | | |
| **SDQ SLOPE - PAC** | | | | |
|  | MAC1 | MAC2 | MAC3 | MAC4 |
| MAC1 | -0.05 (0.16) | . | . | . |
| MAC2 | -0.01 (0.01) | -0.03 (0.16) | . | . |
| MAC3 | -0.02 (0.01) | -0.01 (0.01) | -0.03 (0.16) | . |
| MAC4 | 0.03 (0.07) | 0.04 (0.06) | 0.05 (0.06) | -0.07 (0.17) |

Note. Intercept and slope values for each MAC group are presented on the diagonal. Pairwise comparisons between respective groups are in the remaining cells. Standard error of the estimates is shown in parentheses. * Denotes tests significant at *p*=.05, and ** at *p*=.01.

| Table S17. Coefficients and Their Pairwise Comparisons for the Strengths and Difficulties (SDQ) Prosocial Trajectory Unadjusted Model (Paternal Age Categories [PAC] Only), Full Sample | | | | | |
| --- | --- | --- | --- | --- | --- |
| **SDQ PROSOCIAL TRAJECTORY – full sample** | | | | | |
| **UNADJUSTED MODEL (PAC)** | | | | | |
| **INTERCEPT (PAC)** | | | | | |
|  | PAC1 | PAC2 | PAC3 | PAC4 | PAC5 |
| PAC1 | 7.61 (0.09) | . | . | . | . |
| PAC2 | 0.18 (0.09) | 7.43 (0.02) | . | . | . |
| PAC3 | 0.26 (0.09)* | 0.08 (0.04) | 7.35 (0.03) | . | . |
| PAC4 | 0.25 (0.14) | 0.07 (0.11) | -0.01 (0.11) | 7.36 (0.11) | . |
| PAC5 | -0.46 (0.23) | -0.64 (0.21)* | -0.71 (0.21)** | -0.71 (0.24)* | 8.07 (0.21) |
|  | | | | | |
| **SLOPE (PAC)** | | | | | |
|  | PAC1 | PAC2 | PAC3 | PAC4 | PAC5 |
| PAC1 | 0.05 (0.01) | . | . | . | . |
| PAC2 | -0.02 (0.01) | 0.05 (<0.01) | . | . | . |
| PAC3 | -0.02 (0.01) | < -0.01 (< - 0.01) | 0.07 (<0.02) | . | . |
| PAC4 | -0.07 (0.01) | 0.01 (0.01) | 0.02 (0.01) | 0.06 (0.01) | . |
| PAC5 | 0.05 (0.02) | 0.06 (0.02) | 0.07 (0.02) | 0.05 (0.02) | 0.01 (0.02) |
| n | 940 | 11,887 | 6,681 | 628 | 165 |

Note. Intercept and slope values for each PAC group are presented on the diagonal. Pairwise comparisons between respective groups are in the remaining cells. Standard error of the estimates is shown in parentheses.

| Table S18. Coefficients and Their Pairwise Comparisons for the Strengths and Difficulties Questionnaire (SDQ) Prosocial Trajectory in a Fully Adjusted Model (Paternal [PAC] and Maternal [MAC] Age Categories, Offspring Sex and Zygosity and Family’s Socioeconomic Status [SES]) | | | | | |
| --- | --- | --- | --- | --- | --- |
| **SDQ PROSOCIAL TRAJECTORY – full sample** | | | | | |
| **ADJUSTED MODEL (SEX, SES, MATERNAL AGE, ZYGOSITY)** | | | | | |
| **SDQ INTERCEPT - PAC** | | | | | |
|  | PAC1 | PAC2 | PAC3 | PAC4 | PAC5 |
| PAC1 | 7.76 (0.16) | . | . | . | . |
| PAC2 | 0.15 (0.10) | 7.61 (0.13) | . | . | . |
| PAC3 | 0.17 (0.11) | 0.02 (0.05) | 7.59 (0.13) | . | . |
| PAC4 | 0.12 (0.15) | -0.03 (0.01) | -0.05 (0.11) | 7.63 (0.16) | . |
| PAC5 | -0.61 (0.24) | -0.76 (0.22)** | -0.78 (0.22)** | -0.74 (0.24)* | 8.38 (0.24) |
|  | | | | | |
| **SDQ SLOPE - PAC** | | | | | |
|  | PAC1 | PAC2 | PAC3 | PAC4 | PAC5 |
| PAC1 | 0.05 (0.01) | . | . | . | . |
| PAC2 | -0.01 (0.01) | 0.06 (0.01) | . | . | . |
| PAC3 | -0.02 (0.01) | <-0.01 (<0.01) | 0.06 (0.01) | . | . |
| PAC4 | <0.01 (0.01) | 0.02 (0.01) | 0.02 (0.01) | 0.04 (0.01) | . |
| PAC5 | 0.06 (0.02)* | 0.07 (0.02)*** | 0.07 (0.02)*** | 0.05 (0.02)* | -0.01 (0.02) |
| n | 850 | 10557 | 5819 | 550 | 140 |

Note. Intercept and slope values for each PAC/MAC group are presented on the diagonal. Pairwise comparisons between respective groups are in the remaining cells. Standard error of the estimates is shown in parentheses. * Tests significant at *p*=.05, and ** at *p*=.01.

| Table S19. Tetrachoric Correlations for the Intercept and Slope Coefficients in the Strengths and Difficulties Questionnaire (SDQ) Growth Curves | | |
| --- | --- | --- |
|  | **SDQ** | |
|  | **MZ** | **DZ** |
| **Intercept** | 0.67 (0.65-0.70) | 0.32 (0.30-0.34) |
| **Slope** | 0.70 (0.67-0.72) | 0.44 (0.42-0.48) |
| Note: The estimates are given with their 95% confidence intervals (in parentheses). DZ = dizygotic; MZ = monozygotic. | | |

| Table S20. Additive Genetics, Common Environment and Unique Environment Model for the Strengths and Difficulties Questionnaire Coefficients | | |
| --- | --- | --- |
|  | **intercept** | **slope** |
| **h^2^** | 0.53 (0.08-0.70) | 0.48 (0.07-0.73) |
| **c^2^** | 0.05 (0 – 0.43) | 0.15 (0 – 0.49) |
| **e^2^** | 0.41 (0.30-0.56) | 0.37 (0.26-0.51) |
